# Treatment and referral patterns for psoriasis in United Kingdom primary care: a retrospective cohort study

**DOI:** 10.1186/1471-5945-13-9

**Published:** 2013-08-19

**Authors:** Javaria Mona Khalid, Gary Globe, Kathleen M Fox, Dina Chau, Andrew Maguire, Chio-Fang Chiou

**Affiliations:** 1 United Biosource Corporation, London, UK; 2 Amgen Inc., Thousand Oaks, CA, USA; 3 Strategic Healthcare Solutions, LLC, PO Box 543, Monkton, MD 21111, USA; 4 Janssen Global Services, Companies of Johnson & Johnson, New Jersey, USA

**Keywords:** Primary care, General practice, Psoriasis, Referral patterns, UK

## Abstract

**Background:**

In the UK, referrals to specialists are initiated by general practitioners (GPs). Study objectives were to estimate the incidence of diagnosed psoriasis in the UK and identify factors associated with GP referrals to dermatologists.

**Methods:**

Newly diagnosed patients with psoriasis were identified in The Health Improvement Network (THIN) database between 01 July 2007-31 Oct 2009. Incidence of diagnosed psoriasis was calculated using the number of new psoriasis patients in 2008 and the mid-year total patient count for THIN in 2008. A nested case–control design and conditional logistic regression were used to identify factors associated with referral.

**Results:**

Incidence rate of diagnosed adult psoriasis in 2008 was 28/10,000 person-years. Referral rate to dermatologists was 18.1 (17.3-18.9) per 100 person-years. In the referred cohort (N=1,950), 61% were referred within 30 days of diagnosis and their median time to referral was 0 days from diagnosis. For those referred after 30 days (39%, median time to referral: 5.6 months), an increase in the number of GP visits prior to referral increased the likelihood of referral (OR=1.87 95% CI:1.73-2.01). A prescription of topical agents such as vitamin D3 analogues 30 days before referral increased the likelihood of being referred (OR=4.67 95% CI: 2.78-7.84), as did corticosteroids (OR=2.45 95% CI: 1.45-4.07) and tar products (OR=1.95 95% CI: 1.02-3.75).

**Conclusions:**

Estimates of the incidence of diagnosed adult psoriasis, referral rates to dermatologists, and characteristics of referred patients may assist in understanding the burden on the UK healthcare system and managing this population in primary and secondary care.

## Background

Psoriasis is a serious chronic inflammatory skin disease with varying degrees of severity and disability. The most common type, accounting for 90% of all cases, is plaque psoriasis [1]. In the United Kingdom (UK), the prevalence of psoriasis has been reported to be between 0.24% - 1.5%, [2,3]. Higher mortality rates have been reported for severe psoriasis (patients with history of systemic therapy) in the UK [4] and hospital admission rate for patients with a primary diagnosis of psoriasis has been reported to be 2.9 per 10,000 population per year [2]. Psoriasis causes physical impairment, pain, and psychological stress, including impairment in social settings and workplace, and poor health-related quality of life [5-9].

In the UK, the National Institute for Health and Clinical Excellence (NICE) and the British Association of Dermatologists (BAD) treatment guidelines for psoriasis [10,11] recommend starting with prescription topical drugs including corticosteroids, coal tar, dithranol, retinoids (e.g. tazarotene), and vitamin D3 analogues (calcipotriol, calciotriol, tacalcitol) for mild to moderate psoriasis, and progressing to more potent therapies (phototherapy, systemic drugs including methotrexate, ciclosporin, acitretin, and hydroxycarbamide) for greater severity of psoriasis. Anti-tumor necrosis factor agents (e.g. etanercept, adalimumab, infliximab) are indicated in the UK for the treatment of severe plaque psoriasis among patients who do not respond, or are intolerant to or contraindicated for standard systemic therapy [10-12]. At the time of this analysis, the British National Formulary (BNF) restricted the prescribing of systemic biologic drugs acting on the immune system to specialists.

To comprehensively understand psoriasis patients in the UK, the treatment patterns and characteristics of patients treated in primary care before they are referred to dermatologists needs to be elucidated. A previous study reported the number of prescriptions received by patients undergoing therapy [3]. However, data on psoriasis treatment patterns in primary care, especially for patients who are referred to specialist care, is lacking. Additionally, the most current estimates for the incidence of psoriasis in the UK utilized data for patients diagnosed about 15 years ago, in 1996–1997 [13]. It is important to understand the proportion and characteristics of patients who are currently being referred for specialist care to determine an updated disease burden on the UK health system. The objectives of the present study are to estimate the incidence of diagnosed psoriasis and describe the clinical characteristics and treatment patterns for incident psoriasis patients being referred to specialist (dermatologist) care.

## Methods

A retrospective cohort study using The Health Improvement Network (THIN) data to identify patients with a new diagnosis between 1 July 2007 and 31 October 2009 was conducted. The THIN database contains anonymized medical records from 1500 GPs in over 427 primary care practices covering over 5% of the UK population (>7.6 million patients), which is broadly representative of the non-institutionalized UK population [14,15]. Approximately 98% of the UK population is registered with a GP. Diagnoses are recorded using the READ diagnostic code scheme and prescriptions are recorded using codes from the UK Prescription Pricing Authority [16]. THIN is the only UK primary care database that has complete mortality reporting indicated as the acceptable mortality reporting date [17] to ensure the completeness and accuracy of the enumeration of the total population during the study period. THIN data have been previously validated for the study of psoriasis in a population-based setting [18]. The study was reviewed and approved by the Cegedim Strategic Data Medical Research Scientific Review Committee (SRC) (Reference no. 10–035).

### Study population

The study population was comprised of patients in THIN with a diagnosis of psoriasis and who were currently managed by their general practitioner (GP). Newly diagnosed psoriasis patients aged ≥18 years were identified by the first psoriasis Read Code (diagnosis code) in the medical records after 1 July 2007. The index date was defined as the date of first psoriasis diagnosis. Patients were required to be registered with the GP practice for at least one day in the follow-up period (minimum follow-up). Twelve months of computerized records for each patient were required before the first diagnosis of psoriasis to ensure that true incident cases were identified. Patients were excluded if their diagnosis date was earlier than the practice’s acceptable mortality reporting date to increase accuracy of the denominator in the incidence calculations. Follow-up was defined as the time from index date until the earliest of the following: 1) end of study period (i.e. 31 October 2009), 2) date patient left the practice, or 3) date of patient’s death.

### Study measures

Primary outcomes included incidence of diagnosed psoriasis in 2008, and the proportion, characteristics, and treatment patterns of patients referred to specialist (dermatologist) care between 1 July 2007 and 31 October 2009. Incidence of diagnosed psoriasis was defined as the number of new cases of psoriasis in 2008 with a denominator of the mid-year (June) patient count for THIN in 2008. For the incidence person-time calculation, prevalent cases of psoriasis were subtracted from the denominator, and age- and sex-specific rates were calculated from which a UK standardized rate was estimated for 2008.

Patient characterization of the incident cohort referred to dermatologist care included age at diagnosis, gender, body mass index (BMI), select comorbid conditions prior to index date (including hypertension, cardiovascular disease, chronic obstructive pulmonary disease, hyperlipidemia, diabetes mellitus, heart failure, renal insufficiency, cancer, psoriatic arthritis, liver disease, eczema, rheumatoid arthritis, other auto-immune conditions), social class, number of GP visits, and current psoriasis therapy. Psoriasis-related therapies included prescriptions at the index date (or within 30 days after the diagnosis) as well as therapies received during follow-up before a referral to a dermatologist. Social class was represented by the Townsend deprivation index, a census-based index of material deprivation [19].

For identifying factors associated with referral from the GP to dermatologists, a nested case–control design was used. Patients with psoriasis who were not referred to a dermatologist during follow-up were designated as controls. Patients referred to a dermatologist were matched to four controls by GP practice. Matched (by GP practice only) controls were assigned the date of referral of their matched case (referred patients) to investigate potential determinants of referral to a dermatologist.

### Statistical analysis

Referral rates to dermatologists were expressed using survival analysis techniques which accounted for varying lengths of follow-up. Patients that were not referred were censored at the end of their follow-up. Kaplan-Meier survival method was used to calculate the time to referral. Conditional logistic regression was used to calculate adjusted odds ratios (OR) to identify factors associated with referral. The multivariate model was adjusted for age, gender, and variables that were significantly different between groups in an initial bivariate analysis.

## Results

A total of 10,832 patients who were newly diagnosed with psoriasis between 1 July 2007 and 31 October 2009 and met the study inclusion and exclusion criteria were identified. Psoriasis was diagnosed around the age of 49 years for men and women (Table 1). BMI was not significantly different between males and females and its distribution was similar to the general UK population (28 kg/m^2^) [20]. Eczema, present in 31% of newly diagnosed psoriasis patients, was the most commonly reported comorbid condition followed by hypertension (24%). Approximately 40% of patients did not have any of the selected comorbid conditions. The distribution of social class (Townsend deprivation quintiles) was similar to the social class distribution of the general population in THIN. At index date, approximately 80% of newly diagnosed psoriasis patients received topical pharmacological therapy for the treatment of psoriasis. Approximately, 24% of patients started therapy with a combination of vitamin D3 analogues, corticosteroids, and tar or tar combination products, with a total of 40% using vitamin D3 analogues, 39% using corticosteroids, and 14% using tar or tar combination products at index. All other therapies for psoriasis were used by < 1% of patients at index.

**Table 1:** Characteristics of incident psoriasis patients at diagnosis, n = 10,832

**Characteristics**	**Males (n = 5281)**	**Females (n = 5551)**	**All (n = 10,832)**
Age, years (mean, SD)	48.5 (17.1)*	49.4 (18.3)	49.0 (17.8)
Body mass index, kg/m^2^ (mean, SD)	27.4 (5.3)	27.6 (6.4)	27.5 (5.9)
Comorbidities at index, %			
Hypertension	24.1	24.6	24.3
Cardiovascular disease	10.7	7.7	9.1
Chronic obstructive pulmonary disease	7.0	7.2	7.1
Diabetes mellitus	7.5	6.8	7.1
Heart failure	1.2	0.8	1.0
Cancer	4.3	6.3	5.3
Rheumatoid arthritis	4.4	6.3	5.4
Psoriatic arthritis	2.3	2.6	2.4
Eczema	29.0	34.1	31.6
Other autoimmune diseases†	5.5	11.4	8.5
Liver disease	0.04	0.2	0.1
Dyslipidemia	9.4	8.1	8.7
Number of comorbid conditions, %			
0	43.7	37.4	40.5
1	31.9	33.0	32.5
2 or more	24.4	29.6	27.1
Townsend deprivation quintile, %			
1	23.9	22.6	23.3
2	20.7	20.4	20.6
3	21.0	21.2	21.1
4	16.9	17.6	17.3
5	13.0	13.4	13.2
Psoriasis-related therapies at index, %			
Vitamin D3 analogues	45.5*	34.7	39.9
Retinoids	0.04	0.07	0.06
Tar & tar combination products	12.0	15.9	14.0
Anthracin & combination products	0.6	0.5	0.6
Salicylic acid	0.8	0.8	0.8
Corticosteroids	34.9	43.6	39.4
Phototherapy	0	0.04	0.02
Non-biologic systemic therapy	0.6	0.8	0.7
Any psoriasis-related medication at index, %	80.6	80.1	80.4

†includes ankylosing spondylitis, Crohn’s disease, irritable bowel syndrome, metabolic syndrome, ulcerative colitis.

*P<0.05.

### Incidence of diagnosed psoriasis

The standardized incidence rate of diagnosed adult psoriasis in 2008 was 28 (95% CI: 28–29) per 10,000 person-years. The incidence rate was highest for 60–69 year olds at 34 (95% CI: 31–36) per 10,000 person-years. Incidence did not vary by sex (Table 2).

**Table 2:** Incidence rates of diagnosed psoriasis for 2008 by age and sex per 100 person-years

	**Incidence rate per 100 person-years (95% CI)**	
**Age group, years**	**Males**	**Females**
18-29	0.25 (0.23-0.28)	0.35 (0.32-0.38)
30-39	0.29 (0.26-0.32)	0.32 (0.28-0.35)
40-49	0.22 (0.20-0.25)	0.22 (0.20-0.24)
50-59	0.32 (0.30-0.36)	0.31 (0.28-0.34)
60-69	0.37 (0.33-0.40)	0.31 (0.28-0.35)
70-79	0.29 (0.25-0.33)	0.29 (0.25-0.32)
80 and older	0.18 (0.14-0.23)	0.16 (0.13-0.19)

### Referrals to dermatologists

**Figure 1:**
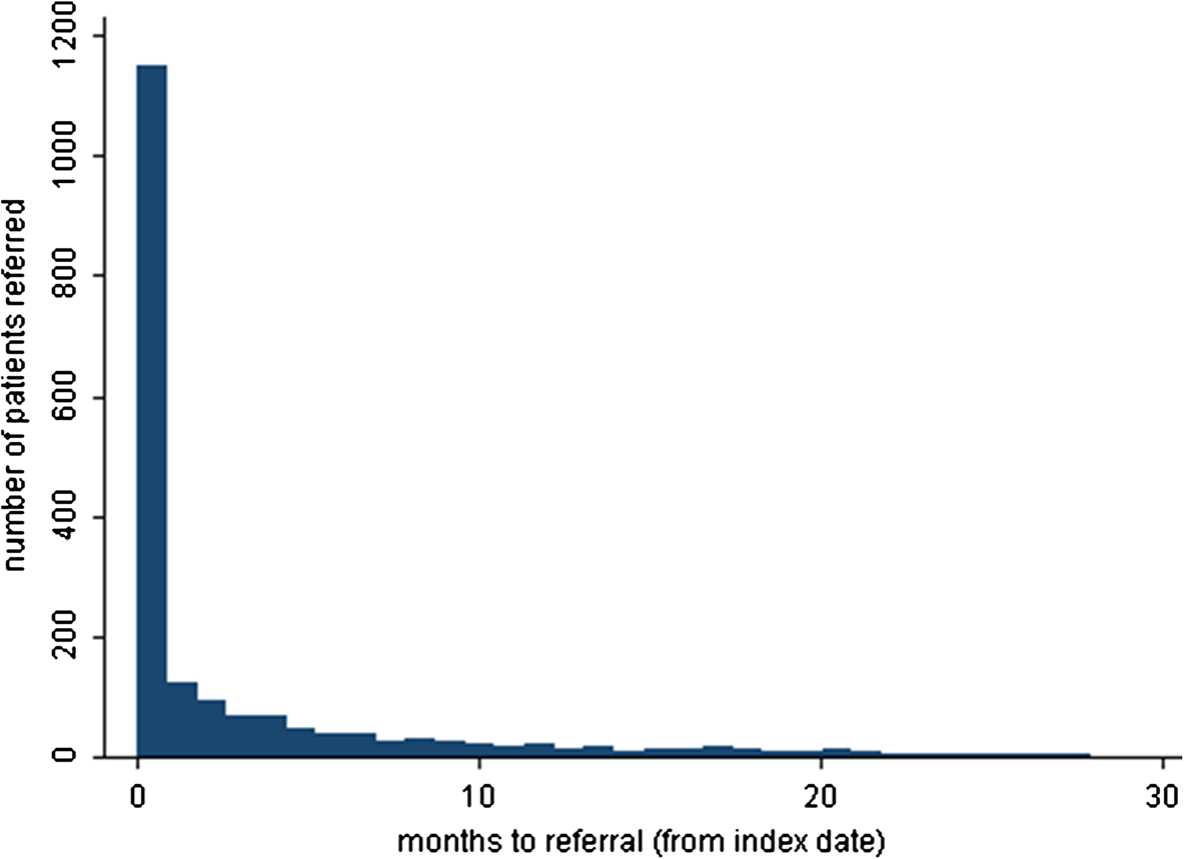
Distribution of time to referral to secondary care dermatology specialist (n = 1,950).

A total of 1,950 (18%) patients within the incident cohort had a referral to a dermatologist. The referral rate was 18.1 (95% CI: 17.3-18.9) per 100 person-years. Amongst those that were referred, 49% were male and median age at diagnosis was 49 (interquartile range: 35–62) years. The distribution of time to referral showed a clustering of patients who were referred very shortly after diagnosis (within 30 days of diagnosis). For patients referred after 30 days of diagnosis, the distribution of time to referral ranged from 31 days to 2.3 years with no clear clustering so these patients were grouped together as referral >30 days after diagnosis. Approximately 61% of referred patients (n = 1,183) were referred to a dermatologist within 30 days of diagnosis (immediately referred) and 39% were referred after 30 days post-diagnosis and were managed in primary care up to their referral date (Figure 1).

The median time to referral for patients referred within the first month was the day of diagnosis (day 0, interquartile range: 0–6 days). Demographic characteristics, including age (mean [SD]: 49.2 years [17.5] vs. 48.8 [17.9]), gender (46.8% vs. 48.8% men), BMI (28.1 kg/m^2^ [6.4] vs. 27.6 [6.0]), and number of psoriasis-related prescriptions at index (0.6 [0.9] vs. 0.4 [0.7]) were similar between cases that were immediately referred and their matched controls that were not referred to a dermatologist. Mean number of GP visits in the year prior to referral was 2.0 [1.5] for immediately referred patients compared to 1.8 [1.4] for patients not referred to a dermatologist (p<0.05). Mean number of comorbid conditions was 1.2 [1.3] for immediately referred patients compared to 1.1 [1.2] for patients not referred (p<0.05). For those who were referred immediately, a greater number of GP visits prior to a dermatologist referral significantly increased the likelihood of referral (OR = 1.14, 95% CI: 1.08-1.20) compared to controls, after adjusting for age, gender, number of comorbid conditions, psoriatic arthritis diagnosis, and psoriasis-related prescription at index (Table 3). A prescription at index of tar products also increased the likelihood of being referred immediately (OR = 2.00, 95% CI: 1.45-2.76), as did vitamin D3 analogue (OR = 1.84, 95% CI: 1.50-2.25) and corticosteroid (OR = 1.56, 95% CI: 1.26-1.92) prescriptions compared to controls (Table 3).

**Table 3:** Risk of referral from conditional logistic regression for patients referred to a dermatologist

**Variable**	**Immediately referred at diagnosis**	**Referred >30 days after diagnosis**
	**Adjusted† Odds ratio (95% CI)**	**Adjusted† Odds ratio (95% CI)**
Age	1.00 (0.98-1.00)	0.99 (0.98-1.00)
Males	1.07 (0.92-1.23)	0.87 (0.71-1.07)
Number of comorbidities in prior year	1.01 (0.94-1.08)	0.96 (0.87-1.06)
Number of GP visits in prior year	1.14 (1.08-1.20)*	1.87 (1.73-2.01)*
Psoriatic arthritis at index	1.16 (0.62-2.19)	1.15 (0.61-2.16)
No psoriasis-related prescription at index	Reference	NA
Corticosteroid prescription at index	1.56 (1.26-1.92)*	NA
Vitamin D3 analogue prescription at index	1.84 (1.50-2.25)*	NA
Tar & tar combination prescription at index	2.00 (1.45-2.76)*	NA
Non-biologic systemic therapy at index	0.30 (0.11-0.88)*	NA
No psoriasis-related prescription in 30 days prior to referral	NA	Reference
Corticosteroid prescription in 30 days prior to referral	NA	2.43 (1.45-4.07)*
Vitamin D3 analogue prescription in 30 days prior to referral	NA	4.67 (2.78-7.84)*
Tar & tar combination prescription in 30 days prior to referral	NA	1.95 (1.02-3.75)*
Non-biologic systemic therapy in 30 days prior to referral	NA	0.20 (0.05-0.90)*
Number of psoriasis-related prescriptions in 30 days prior to referral	NA	0.85 (0.31-1.17)

†Adjusted for age, gender, number of comorbidities, psoriatic arthritis diagnosis, and psoriasis-related prescriptions at index; *NA* not applicable.

*P<0.05.

For patients who were referred to a dermatologist > 30 days after diagnosis (later referred patients), demographic characteristics, including age (48.5 years [19.0] vs. 49.2 [17.8]), gender (51.1% vs. 50.3% men), BMI (28.1 kg/m^2^ [6.2] vs. 27.6 [6.0]), and the number of comorbid conditions (1.3 [1.3] vs. 1.2 [1.3]) in the year period were similar between cases (later referred patients) and controls. The median time to referral to a dermatologist was 5.6 (interquartile range: 2.8-11.5) months after the psoriasis diagnosis. The mean number of GP visits in the year prior to referral was 3.0 [2.0] for later referred patients compared to 1.4 [1.3] for patients not referred to a dermatologist (p<0.05); the mean number of psoriasis-related prescriptions at index was 0.6 [1.0] for later referred patients and 0.1 [0.5] for controls (p>0.05). Overall, later-referred patients made 5.34 visits to the GP before they were referred to a dermatologist. Of those GP visits, 2.73 visits were psoriasis-related.

After adjusting for age, gender, number of comorbid conditions, psoriatic arthritis diagnosis, and psoriasis-related prescription, a greater number of GP visits prior to referral increased the likelihood of later referral (OR = 1.87, 95% CI: 1.73-2.01) compared to controls (Table 3). A prescription for vitamin D3 analogues within 30 days before referral also increased the likelihood of being later referred (OR = 4.67, 95% CI: 2.78-7.84), as did corticosteroids (OR = 2.43) and tar products (OR = 1.95) compared to controls.

## Discussion

Approximately 28 patients per 10,000 person-years were newly diagnosed with psoriasis in the UK. Among patients who were diagnosed in primary care, the referral rate to dermatology was 18 per 100 person-years, with most patients who were referred being referred immediately after a diagnosis is made. Having a greater number of GP visits in the year prior to referral, a prescription for vitamin D3 analogues, corticosteroids or tar products increased the likelihood of being referred to the dermatologist.

Previous population-based UK studies have reported psoriasis incidence to be lower as 14 per 10,000 person-years [13,21]. One possible explanation of the higher incidence estimate of diagnosed psoriasis in the present study is that previous studies were conducted prior to the introduction of the Quality Outcomes Framework (QOF) guidelines [22], which have improved the completeness of diagnostic recording in UK primary care since payment is performance-related under QOF. An increased awareness of psoriasis and new treatment options may also be a reason for the higher incidence of diagnosed psoriasis in recent years. The bimodal distribution of incidence of diagnosed psoriasis with rates peaking in the 30–39 and 60–69 year age groups in this study was also reported in a previous study of the UK population [13].

In the present study, approximately 18% of newly diagnosed psoriasis patients were referred to dermatologists. In the UK, the first point of contact with health professionals is the GP. The GP may decide to manage psoriasis patients in primary care, especially when the disease is mild, or refer patients to dermatologists in secondary care if the psoriasis is more severe. According to guidelines and the BNF, most systemic therapies including biologics can only be prescribed by a specialist [10-12]. Recent evidence indicates that 25% to 44% of psoriasis patients are moderate to severe and would likely benefit from specialist attention [23-25]. However, the present study found that only 18% of patients were referred, suggesting under-utilization of specialist services. The referral rate of 18% is higher than the previously reported figure of 0.7% in 2003 using data from the Doctor’s Information Network, a smaller primary care database in the UK [21]. The higher rate of referral in the present study may also be explained, in part, by the recent availability (since 2003) of biologics in secondary care and biologics can only be prescribed by a specialist in the UK.

Most patients in the present study were referred immediately after the psoriasis diagnosis. One possible reason is these patients may present with severe psoriasis at the time of diagnosis, necessitating immediate referral to a dermatologist.

A greater number of GP visits in the year prior to referral, a prescription for vitamin D3 analogues, corticosteroids or tar products were all significantly associated with an increased likelihood of referral to a dermatologist amongst patients referred immediately or later. The factors associated with referral may help in understanding which patients need specialist care so that in the future, patients may be identified and referred earlier and, where appropriate, receive systemic therapies (biologic and non-biologic) to control their psoriasis. With the association between vitamin D3 analogues, corticosteroids and tar products and referral to a dermatologist, it appears that GPs are following the UK NICE and BAD guidelines [10,11] for step therapy of starting with prescription topical drugs since corticosteroids and vitamin D3 analogues were most frequently prescribed at index. Also, a greater proportion of referred patients received topical therapies within 30 days prior to referral than at index. Age, gender, BMI, concomitant psoriatic arthritis, and number of comorbid conditions were found to not be associated with an increased likelihood of referral to a dermatologist.

One of the strengths of this study is that THIN is one of the few primary care databases validated to study psoriasis [18]. Furthermore, as a population-based database was used, the results from this study are broadly representative of the UK population in terms of age and gender. The study data are presented for a recent time period, after the introduction of incentivized guidelines on completeness of coding in primary care and represent estimates from a period where GPs may be coding more accurately.

The study does have some limitations. It was not possible to directly discern severity of the psoriasis amongst the patient population since medical Read codes do not consistently indicate severity of the condition. It is possible that patients with mild psoriasis may not have come to medical attention in which case diagnoses by GPs may underestimate the incidence of psoriasis. The clinical characteristics and treatment patterns described in this study are those that are prescribed in primary care by GPs and the treatment patterns for systemic therapy are not included in primary care data. Other factors such as psoriasis severity, patient understanding of the disease, and patient desire for improvement, may be associated with GP referrals to dermatologists; however, data for these factors were not recorded or available in the THIN database.

## Conclusion

In conclusion, the current estimates of the magnitude of the diagnosed psoriasis population and referral rates to dermatologists may assist in understanding the burden on the UK healthcare system and allow for decision-making and planning for managing this chronic disease population in primary and secondary care.

## Consent

The THIN database contains only anonymized and de-identified clinical data and no direct patient contact occurred in this retrospective study. As such, this study was exempt from obtaining patient consent.

## Abbreviations

BAD: British association of dermatologists; BMI: Body mass index; BNF: British national formulary; GP: General practitioners; NICE: National institute for health and clinical excellence; OR: Odds ratio; QOF: Quality outcomes framework; THIN: The health improvement network; UK: United Kingdom.

## Competing interests

Drs. Khalid, Fox, and Maguire received research funds from Amgen, Inc. Drs. Globe and Chau are employed by Amgen, Inc. and are stockholders. Dr. Chiou was employed by Amgen, Inc. at the time of this research.

## Authors’ contributions

JMK oversaw the data acquisition, conducted the data analysis and interpretation, performed the statistical testing, provided critical review and revision of the article, and approved of the final version of the article. GG contributed significantly to the study concept and study design, provided data interpretation, provided critical review and revision of the article, and approved of the final version of the article. KMF contributed significantly to the study concept and study design, provided data interpretation, drafted the article, provided critical revision of the article, and approved of the final version of the article. DC contributed to the data interpretation, provided funding for the study, provided critical review and revision of the article, and approved of the final version of the article. AM contributed significantly to the study concept and study design, provided data interpretation, provided critical review and revision of the article, and approved of the final version of the article. CC contributed significantly to the study concept and study design, provided data interpretation, provided critical review and revision of the article, and approved of the final version of the article. All authors read and approved the final manuscript.

## Pre-publication history

The pre-publication history for this paper can be accessed here:

http://www.biomedcentral.com/1471-5945/13/9/prepub
